# When Asthma Leads to Air: Pneumomediastinum in a Child

**DOI:** 10.3390/children13070959

**Published:** 2026-07-21

**Authors:** Giuliana Ferrante, Laura Tenero, Eleonora Carlesso, Giorgia Dalla Vista, Stefano Rosa, Michele Piazza, Giorgio Piacentini

**Affiliations:** 1 Pediatric Division, Department of Surgical Sciences, Dentistry, Gynecology and Pediatrics, University of Verona, 37126 Verona, Italy; giuliana.ferrante@univr.it (G.F.); eleonora.carlesso@studenti.univr.it (E.C.); giorgia.dallavista@studenti.univr.it (G.D.V.); stefano.rosa_01@studenti.univr.it (S.R.); giorgio.piacentini@univr.it (G.P.); 2 Pediatric Division, University Hospital of Verona, 37134 Verona, Italy; laura.tenero@aovr.veneto.it

**Keywords:** asthma, children, pneumomediastinum, pneumorrhachis

## Abstract

**Highlights:**

**What are the main findings?**

- Respiratory diseases, particularly asthma, are among the most frequent predisposing conditions to pediatric pneumomediastinum.
- Diagnosis relies on clinical assessment and chest imaging, while management is mainly conservative.

**What are the implications of the main findings?**

- Prompt and accurate recognition, along with appropriate management of underlying respiratory conditions, is essential to prevent complications such as pneumomediastinum and to ensure favorable outcomes.
- Pediatric pneumomediastinum is usually self-limiting when identified early and in the absence of severity criteria. Timely intervention, targeted therapy, and close follow-up are critical, allowing most children to recover fully without the need for invasive procedures.

**Abstract:**

**Background:** Pneumomediastinum is a clinical condition characterized by the presence of free air within the mediastinum. **Methods**: We report the case of a 12-year-old boy with recurrent wheezing who developed pneumomediastinum associated with pneumorrhachis during a Rhinovirus-triggered asthma exacerbation, in the context of a family history of spontaneous pneumothorax, alongside a literature review covering the past ten years. **Results**: Laboratory tests were unremarkable, except for neutrophilia and a positive Rhinovirus test. Chest X-ray revealed a large pneumomediastinum, extending into cervical soft tissues, and CT confirmed pneumorrhachis. The patient was treated with oxygen, bronchodilators, corticosteroids, and antibiotics due to concern for esophageal injury, and he was discharged after six days with complete resolution on follow-up. Pediatric pneumomediastinum is rare but usually benign. Respiratory diseases, particularly asthma, are among the most frequent predisposing conditions. Diagnosis relies on clinical assessment and chest imaging, while management is mainly conservative. **Conclusions**: Timely intervention is essential in the care of children with pneumomediastinum. Accurate diagnosis and optimal management of asthma are essential not only to control symptoms but also to prevent potential serious complications. This case highlights the occurrence of pneumorrhachis as an uncommon radiological finding associated with asthma-related pneumomediastinum and provides an updated review of recent pediatric evidence.

## 1. Introduction

Pneumomediastinum is a rare and generally benign, self-limiting clinical condition in children, characterized by the presence of free air within the mediastinum. This air can then extend to the hilum and surrounding mediastinal structures. Once in the mediastinum, it may further track along tissue planes, spreading to the neck, face, abdomen, or even the limbs, resulting in subcutaneous emphysema. The presence of air within the spinal canal is termed pneumorrhachis [1]. Reported incidence in pediatric populations varies widely, ranging from 1 in 800 to 1 in 42,000 patients presenting to emergency departments [2].

In children, pneumomediastinum is generally classified as either traumatic/iatrogenic or non-traumatic. Traumatic and iatrogenic causes—such as chest trauma, mechanical ventilation, endoscopic procedures, and thoracic surgery—are less common in children than in adults. Non-traumatic pneumomediastinum, the most frequent form, is typically referred to as spontaneous pneumomediastinum in the literature. It may occur secondary to underlying respiratory or gastrointestinal conditions, or it may be idiopathic, often associated with Valsalva-like maneuvers, including coughing, vomiting, crying, shouting, or intense physical exertion [2,3,4]. Respiratory conditions, including asthma, pulmonary infections, cystic fibrosis, and foreign body aspiration, are among the most common predisposing conditions associated with pediatric pneumomediastinum. Rather than representing direct causes, these conditions promote increases in intra-alveolar pressure, leading to alveolar rupture and air dissection along the peribronchovascular sheaths. Gastrointestinal causes, particularly esophageal rupture (usually following vomiting), can also lead to pneumomediastinum with a distinct mechanism by which air directly enters the mediastinum [5]. Nevertheless, spontaneous pneumomediastinum in children is likely underdiagnosed [6].

Management of pneumomediastinum is primarily supportive, focusing on addressing the underlying cause. The typical hospital stay averages 1–2 days, reflecting the generally benign and self-limiting course of the condition [7]. Potential complications include extrinsic airway compression due to mediastinal air accumulation, which may result in respiratory distress and/or dysphagia; cardiac tamponade, also caused by extrinsic compression and potentially manifesting with signs of heart failure; and mediastinitis, a serious infection requiring prompt antimicrobial therapy [1].

We report the case of a 12-year-old boy with a history of recurrent wheezing who presented with pneumomediastinum complicated by pneumorrhachis during a Rhinovirus-associated asthma exacerbation (Table 1). The coexistence of pneumorrhachis, viral-triggered asthma, and a family history of spontaneous pneumothorax makes this case clinically noteworthy. Additionally, a narrative review of the literature was performed using the PubMed database. The search strategy combined the terms “child”, “pneumomediastinum”, “asthma”, and “pneumorrhachis” using different Boolean operators: ((child) AND (pneumomediastinum) AND (asthma)), (pneumomediastinum) AND (asthma), and (pneumomediastinum) AND (pneumorrhachis) AND (asthma). The search included articles published between 1 January 2016 and January 2026. Age limits were set to children (birth–18 years), and no restrictions were placed on study location. Titles and abstracts were screened for relevance (n = 66). Non-English publications, studies involving adults, traumatic or iatrogenic pneumomediastinum, were excluded (n = 29). Original articles, reviews, and case reports reporting pediatric patients with pneumomediastinum associated with asthma, with or without pneumorrhachis, were included (n = 15). Reference lists of retrieved articles were also consulted (n = 11). The studies showed in Table 2 were case reports selected because they fulfilled the predefined eligibility criteria and provided sufficient clinical information for comparison with our case.

## 2. Case Report

A 12-year-old boy was admitted to the emergency department with dyspnea and chest pain. He had experienced a single episode of vomiting but no fever. The main clinical features are summarized in Table 1.

**Table 1:** Main features of the case report.

**Sex**	Male
**Age**	12 years
**Vital signs**	HR 102 beats/min, BP 110/60 mmHg, RR 40/min, SpO_2_ 99% on 1–2 L/min supplemental O_2_ via nasal cannula, T 36.5 °C
**Symptoms**	Difficulty in breathing and chest pain. One episode of vomiting; no fever
**Past medical history**	Allergic rhino-conjunctivitis and recurrent bronchospasm.
**Family history**	Spontaneous pneumothorax in his father at 18 years of age.
**Risk factors**	Recurrent wheezing
**Venous blood gas analysis**	pH 7.37, pCO_2_ 40 mmHg, HCO_3_ 23 mmol/L, BE −2, lactate 2.7 mmol/L
**WBC on hospital admission**	12,890/mm^3^ (NR 4500–13,000)
**C-Reactive Protein** **on hospital admission**	3 mg/L (NR < 5)
**Alpha-1 antitrypsin level**	1.47 g/L (NR 0.9–2)
**Respiratory viral panel on** **nasopharyngeal aspirate**	Rhinovirus
**Chest X-ray**	Pneumomediastinum with extension into the bilateral later cervical soft tissues and the chest wall, more pronounced on the left No pleural effusion. No parenchymal lesions
**Chest CT**	Hypodense material in the subsegmental bronchial branches of the lower lobes No pleural effusion Marked pneumomediastinum with subcutaneous emphysema involving the soft tissues of the neck and the proximal portions of the chest wall and back Air foci within the spinal canal
**Therapy**	Oxygen, oral prednisone, inhaled salbutamol, ipratropium and beclomethasone Oral Amoxicillin/Clavulanic Acid
**Therapy at discharge**	Salmeterol/Fluticasone propionate 25/50 mcg, 2 inhalations twice daily
**Chest X-ray at follow-up**	Resolution

HR: heart rate; BP: blood pressure; RR: respiratory rate; WBCs: White blood cells; NR: normal reference; CT: computer tomography.

The patient had a history of allergic rhino-conjunctivitis and recurrent wheezing, treated only on an as-needed basis. His family history was notable for a spontaneous pneumothorax in his father at 18 years of age.

On arrival, the patient demonstrated oxygen desaturation (SpO_2_ 93–94%), and supplemental oxygen at 2 L/min via nasal cannula was administered. Arterial blood gas analysis showed normal compensation and adequate gas exchange (pH 7.37, pCO_2_ 40 mmHg, HCO_3_ 23 mmol/L, BE −2, lactate 2.7 mmol/L). Laboratory tests revealed a normal leukocyte count (12,890/mm^3^) with elevated neutrophils (11,960/mm^3^, which was considered a nonspecific finding, likely reflecting the acute physiological stress associated with the respiratory exacerbation), while hemoglobin and platelet levels were within normal limits. Additional testing showed normal C-reactive protein and alpha-1 antitrypsin levels. Nasopharyngeal aspirate tested positive for Rhinovirus infection.

Chest X-ray (Figure 1) revealed a large pneumomediastinum extending into the soft tissues of both lateral cervical compartments and the thoracic wall, more pronounced on the left side. No pleural effusion or lung parenchymal abnormalities were noted.

The patient was transferred to our tertiary care department. On examination, he appeared in good general condition. Chest auscultation revealed markedly diminished air entry with widespread expiratory wheezing. Palpable subcutaneous crepitus was present in the cervical, axillary, and thoracic regions, consistent with subcutaneous emphysema.

Treatment was initiated with aerosol therapy including salbutamol and ipratropium, along with oral prednisone. He was subsequently admitted to the pediatric ward for ongoing care and further evaluation. Vital signs on admission were as follows: heart rate 102 beats/min, blood pressure 110/60 mmHg, respiratory rate 40 breaths/min, oxygen saturation 99% on 1–2 L/min supplemental oxygen via nasal cannula, and pain score 3/10 (Numeric Rating Scale). The patient was alert and afebrile. Chest auscultation confirmed reduced bilateral air entry with diffuse bilateral polyphonic expiratory wheezing. Oxygen therapy was continued for approximately 72 h. Inhaled therapy was delivered by nebulization and consisted of salbutamol (2.5 mg every 6 h) and beclomethasone dipropionate (400 μg every 8 h). Oral prednisone was administered at a dose of 25 mg twice daily for the first 72 h. Clinical status, vital signs, and pain levels were closely monitored, and analgesics were provided as needed.

Given the vomiting episode at home and concern for potential esophageal injury, a chest computed tomography (CT) scan was performed, and oral amoxicillin–clavulanic acid was started. Chest CT (Figure 2) revealed hypodense material within the subsegmental bronchi of the lower lobes, most likely representing retained bronchial secretions, without associated atelectasis or parenchymal consolidation. Cardiac size and morphology were normal, with no pleural or pericardial effusion. A pronounced pneumomediastinum was confirmed, with emphysema of the soft tissues of the neck and proximal thoracic wall and back (Figure 3). Small gas collections were also observed within the spinal canal (Figure 4).

Antibiotic therapy was subsequently discontinued once esophageal perforation had been ruled out. Oxygen therapy was discontinued following sustained clinical improvement, characterized by stable vital signs and marked reduction in the subcutaneous emphysema. As the patient’s respiratory status improved, nebulized therapy and oral prednisone were progressively tapered before being discontinued. The patient was discharged after six days of hospitalization. He was advised to begin maintenance inhalation therapy with salmeterol–fluticasone 25/50 µg (2 inhalations twice daily) via pMDI and a valved holding chamber. A follow-up chest X-ray performed seven days after discharge demonstrated complete resolution.

## 3. Discussion

Pneumomediastinum is a rare occurrence in children. Consistent with our case, adolescent males are most frequently affected [7,8]. The mean age at presentation ranges from 6.9 to 14 years [4], with some evidence suggesting a bimodal peak in incidence—occurring in children younger than four years and in adolescents aged 15–18 years [9]. A pronounced sex disparity is also reported, with males representing 77.7% of cases compared to 22.2% in females (Table 2) [10,11,12,13,14,15,16,17].

Our case is noteworthy for several reasons. First, pneumorrhachis is an uncommon radiological finding in children with spontaneous pneumomediastinum. Second, the episode occurred during a Rhinovirus-associated asthma exacerbation in a child with recurrent wheezing, further supporting asthma as the underlying mechanism. Third, the presence of a family history of spontaneous pneumothorax prompted consideration of an inherited predisposition, although no clinical features suggestive of an underlying connective tissue disorder were identified. Finally, we complement this case with an updated review of the pediatric literature from the last decade.

Data in the literature are inconsistent regarding the proportion of pneumomediastinum cases associated with a precipitating event. Some studies report that 70–90% of cases have an identifiable trigger [9], whereas others suggest that only 40–60% of cases are linked to a precipitating factor [18,19]. Respiratory diseases, such as asthma exacerbations and lower respiratory tract infections, are among the most common predisposing conditions, predominantly affecting adolescents and children under six years of age, respectively [5]. Foreign body aspiration should also be considered, particularly in younger children and preschoolers [19]. In our literature review, 55.5% of patients had a prior diagnosis of asthma. Among those without a previous asthma diagnosis, one patient had a history of recurrent wheezing, as in our case; two experienced wheezing for the first time; and one had exertional dyspnea. Additionally, some cases reported exposure to cigarette smoke or environmental smoke following recent fires.

The pathophysiological mechanism most commonly implicated is the Macklin effect, which consists of alveolar rupture caused by a sudden increase in intra-alveolar pressure, followed by air dissection along the peribronchovascular sheaths toward the mediastinum [20].

Pain is the most commonly reported symptom of pneumomediastinum, typically presenting as retrosternal chest pain, neck pain, or sore throat. Dyspnea and cough are also frequent, observed in 67% and 89% of patients, respectively [7]. On physical examination, subcutaneous emphysema (palpable crepitus) is reported in only 10% of cases, whereas neck pain occurs in over 50%. Hamman’s sign—a precordial crunching sound synchronous with the heartbeat—is a highly suggestive clinical finding [21]. Other reported symptoms include wheezing, chest tightness, neck fullness, fever, unusual sensations in the neck and chest, odynophagia, and dysphagia with food refusal.

Laboratory investigations may aid in identifying the underlying cause and typically include blood gas analysis, complete blood count, inflammatory markers such as C-reactive protein, and biochemical or metabolic panels. Alpha-1 antitrypsin (A1AT) deficiency is rarely a cause of pneumomediastinum, so routine measurement is not generally recommended [22]. In our case, A1AT levels were measured because of a family history of spontaneous pneumothorax, as part of the evaluation for potential underlying conditions associated with pulmonary parenchymal fragility. Since levels were within the normal range, in the absence of additional clinical features suggestive of an inherited disorder, no further investigations were considered necessary. Nasopharyngeal aspirates can help detect underlying respiratory tract infections.

Chest X-ray remains the first-line imaging modality. Radiographs may reveal subcutaneous emphysema or indirect signs such as Naclerio’s “V” sign, which indicates the presence of air at the junction between the descending aorta and diaphragm [23]. However, the diagnostic sensitivity of chest X-ray is often limited [24], while chest CT may provide additional diagnostic information by detecting subtle pneumomediastinum, defining the extent of air dissemination, and demonstrating air tracking along the peribronchovascular sheaths (the Macklin effect) [25,26]. Nonetheless, chest CT is not routinely indicated but may be considered in specific situations: persistent suspicion despite a negative X-ray, polytrauma, or symptoms suggestive of esophageal rupture such as vomiting or dysphagia [23,27]. Our report illustrates this approach. Although the vomiting was limited to a single episode, the possibility of esophageal perforation could not be confidently excluded based on the initial clinical assessment. Chest CT was performed to rule out this potentially serious complication. Thus, in agreement with previous reports, our case supports the selective rather than routine use of CT in children with pneumomediastinum.

When air extends into the vertebral canal, the condition is termed pneumorrhachis. This phenomenon is thought to occur from air dissecting along fascial planes from the posterior mediastinum and subsequently through the neural foramina into the epidural space, which lacks a complete fascial barrier. The association between pneumomediastinum and pneumorrhachis remains uncommon in the pediatric population. Pneumorrhachis is usually an incidental radiological finding and is most often asymptomatic. In the absence of neurological deficits or spinal cord compression, it generally follows a benign, self-limiting course and resolves spontaneously, without the need for specific treatment or neurosurgical intervention. Neurological complications, such as sensory radiculopathy, motor radiculopathy and myelopathy, have been reported only rarely and are usually associated with traumatic spinal injuries or spinal surgery procedures rather than spontaneous pneumomediastinum [1,28,29,30]. Our literature review found subcutaneous emphysema in 88.8% of cases and pneumorrhachis in 33.3%. Although follow-up chest X-rays were performed in 55.5% of cases, further imaging is generally unnecessary in children without comorbidities [31]. Our patient remained neurologically intact throughout hospitalization and experienced complete clinical recovery, consistent with the favorable prognosis reported in the pediatric literature.

Chalumeau et al. proposed an algorithm for managing pediatric pneumomediastinum, distinguishing three main categories: idiopathic pneumomediastinum, pneumomediastinum secondary to asthma or other underlying diseases, and pneumomediastinum secondary to esophageal rupture [2]. For cases secondary to an underlying condition, severity criteria should be carefully assessed. These include SpO_2_ < 95%, fever, uncontrolled pain, and poor general condition. Hospitalization and treatment of the underlying cause are recommended when any severity criterion is present, as well as in cases secondary to esophageal rupture [2]. In the absence of severity criteria, outpatient management may be appropriate for idiopathic cases or those secondary to an underlying condition, provided that clinical stability is maintained after at least four hours of observation in the emergency department. Hospitalization is indicated for clinical deterioration or symptom progression [31,32,33]. Unlike many children reported in recent series who can be safely managed as outpatients after a short observation period, our patient fulfilled hospitalization criteria because of oxygen desaturation (SpO_2_ 93–94%), extensive subcutaneous emphysema, and the need to exclude esophageal perforation. Therefore, this case supports current recommendations advocating individualized management based on clinical severity rather than routine admission for all children with pneumomediastinum.

Management, whether inpatient or outpatient, is primarily conservative [34], including rest to reduce respiratory workload, analgesia for pain control, and close monitoring of vital signs. Oxygen therapy may enhance alveolar oxygen partial pressure, creating a diffusion gradient (the so-called nitrogen washout effect) that facilitates resorption of interstitial air. According to our literature review, oxygen was used in 22% of cases, typically at low flow (1–2 L/min via nasal cannula), but robust evidence supporting this practice is lacking [35,36], and its use is generally reserved for children with hypoxemia. In our patient, oxygen therapy was administered because of oxygen desaturation, consistently with the available evidence suggesting that oxygen should be reserved for children with oxygen requirement rather than routinely prescribed to accelerate air resorption. Similarly, antibiotic therapy was reported in 22% of cases, though evidence supporting routine use is weak. Antibiotics are generally reserved for patients with fever, leukocytosis, or elevated inflammatory markers. In suspected or confirmed esophageal rupture, prompt broad-spectrum antibiotics are indicated [23,35,36,37,38]. Our patient differed from the typical indication because he had neither fever nor elevated inflammatory markers. However, empirical amoxicillin–clavulanate was started while esophageal perforation was being excluded. Once this diagnosis was ruled out, antibiotic therapy was subsequently discontinued. Thus, our management reflects a precautionary approach driven by the initial differential diagnosis rather than routine treatment of spontaneous pneumomediastinum. Finally, inhaled bronchodilators and corticosteroids, either systemic or inhaled, should follow standard guidelines in children with poorly controlled asthma [14]. In the five cases we reviewed involving children with asthma, a bronchodilator (e.g., salbutamol) was administered, compared with only 22% of children without a known asthma diagnosis. Our case further supports previous observations that asthma is one of the most frequent underlying conditions associated with pediatric pneumomediastinum. Although the patient had not been previously diagnosed with asthma, his clinical history, examination findings, and response to treatment during hospitalization were consistent with a diagnosis of asthma, supporting the initiation of maintenance inhaled therapy after discharge. Indeed, the presence of recurrent wheezing, widespread expiratory wheezing on admission, and Rhinovirus infection strongly suggest that the asthma exacerbation was the precipitating event, emphasizing the importance of recognizing and adequately treating underlying asthma to prevent recurrence.

**Table 2:** Main findings in children with pneumomediastinum reported in the literature in the last ten years.

	Case 1 [10]	Case 2 [11]	Case 3 [12]	Case 4 [13]	Case 5 [14]	Case 6 [15]	Case 7 [15]	Case 8 [16]	Case 9 [17]
**Age**	14	18	18	16	10	5	8	5	9
**Sex**	Male	Male	Male	Male	Female	Female	Male	Male	Male
**Symptoms**	Wheezing, cough, respiratory distress, crepitus of the neck	Dyspnea, chest tightness, thoracic pain, cough	Dyspnea, chest tightness, cough	“Neck fullness”, cough, wheezing, shortness of breath	Cough, dyspnea, chest/neck pain	Cough, fever	Neck pain, cough	Cough, dyspnea, odynophagia, dysphagia with food refusal and neck pain	Thoracic pain, dyspnea
**Subcutaneous emphysema**	Yes	Yes	Yes	Yes	Yes	Yes	No	Yes	Yes
**Pneumorrhachis**	No	Yes	Yes	No	No	No	No	No	Yes
**History** **of Asthma**	Yes	Yes	Yes	Yes	Yes	No	No	No	No
**Other risk factors**	No	No	Smoking history	No	ETS and ash after a recent fire	First-time wheezing episode	First-time wheezing episode	Recurrent wheezing	Exertional dyspnea
**Chest X-ray**	Yes	Yes	Yes	Yes	Yes	Yes	Yes	Yes	Yes
**Chest CT**	No	Yes	Yes	Yes	No	No	Yes	No	Yes
**Therapy**	Inhaled salbutamol and ipratropium, IV magnesium, and steroids	Inhaled salbutamol, systemic steroids	Inhaled salbutamol and symptomatic treatment	Inhaled salbutamol ipratropium, oral steroids	Inhaled salbutamol and ipratropium, IV steroids	Conservative	Conservative	Analgesia, salbutamol, steroids	Inhaled salbutamol, steroids
**Oxygen**	No	No	No	No	Yes	No	No	Yes	No
**Antibiotics**	No	Yes	No	No	No	No	No	No	Yes
**Chest X-ray at follow-up**	No	No	No, but repeated CT scan 5 days later	Yes	Yes	Yes	Yes	No	Yes
**Length** **of stay**	7 days	1 day	Not reported	Not reported	4 days	7 days	4 days	5 days	Not reported
**Therapy** **at discharge**	Inhaled salbutamol and oral prednisone	ICS and SLIT	Not reported	Not reported	ICS	Not reported	Not reported	Not reported	ICS

ETS: environmental tobacco smoke; ICS: inhaled corticosteroids; IV: intravenous; SLIT: sub-lingual immunotherapy.

## 4. Conclusions

In conclusion, we report the case of a 12-year-old boy with a history of recurrent bronchospasm who presented with pneumomediastinum and pneumorrhachis, alongside a literature review covering the past ten years. Pediatric pneumomediastinum is uncommon but generally benign, most frequently affecting adolescent males. The condition may be idiopathic or secondary to triggers, including asthma exacerbations. Management includes rest, analgesia, treatment of underlying conditions, and close monitoring. Hospitalization is indicated for patients meeting severity criteria, whereas stable children may be safely managed as outpatients. Early recognition, targeted therapy, and vigilant follow-up are key to achieving excellent outcomes, with most children recovering fully without invasive interventions. Open issues in the management of pneumomediastinum regard oxygen and antibiotic treatment; given the rarity of the condition, further studies and international consensus may help to better define and standardize the clinical care. Nonetheless, treatment decisions should be individualized according to the patient’s clinical presentation and differential diagnosis.

This report contributes to the literature by describing the uncommon association of pneumomediastinum with pneumorrhachis occurring during a virus-triggered asthma exacerbation in a child with a family history of spontaneous pneumothorax. Together with our review of recent pediatric cases, it emphasizes the importance of recognizing asthma as a major predisposing factor while also highlighting pneumorrhachis as a rare but usually benign complication requiring no specific treatment.

## Figures and Tables

**Figure 1:**
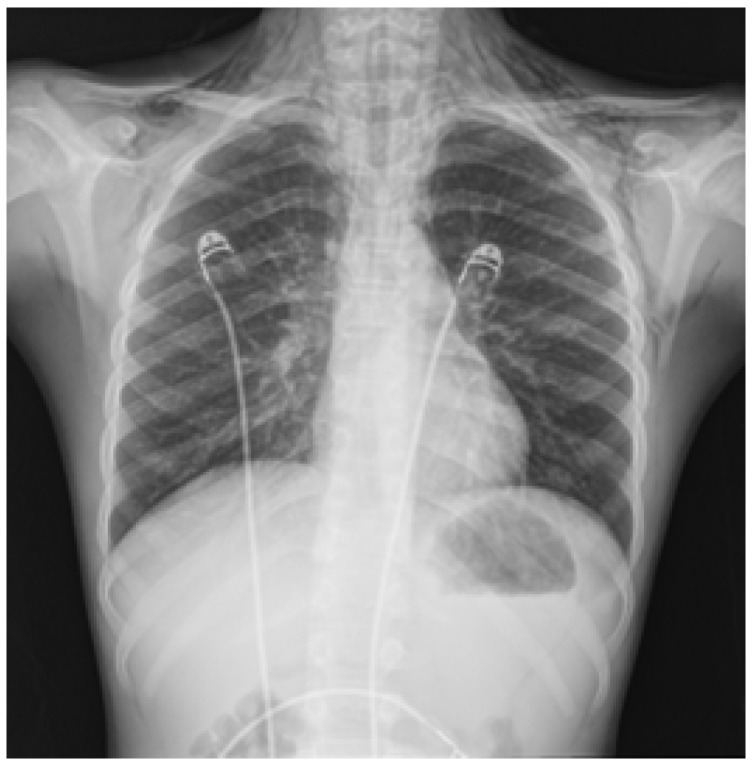
Chest X-ray showing a large pneumomediastinum extending into the soft tissues of both lateral cervical compartments and the thoracic wall.

**Figure 2:**
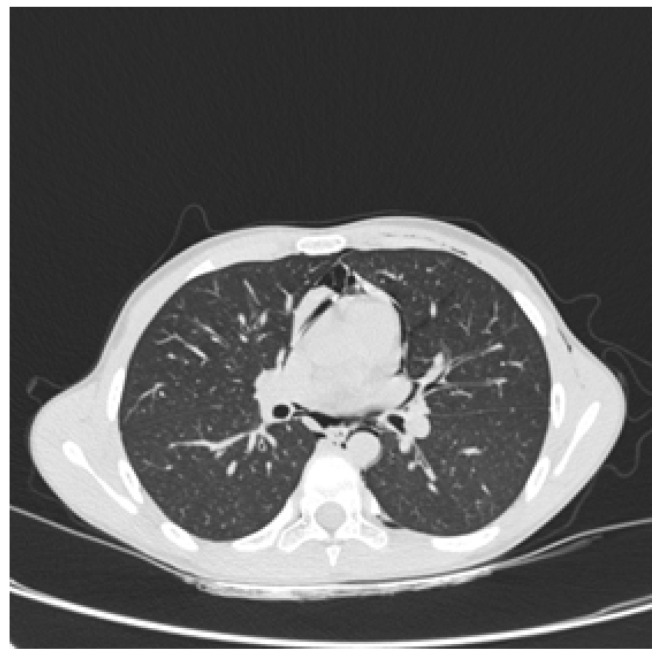
Chest CT (transverse section) showing hypodense material in the subsegmental bronchi of the lower lobes.

**Figure 3:**
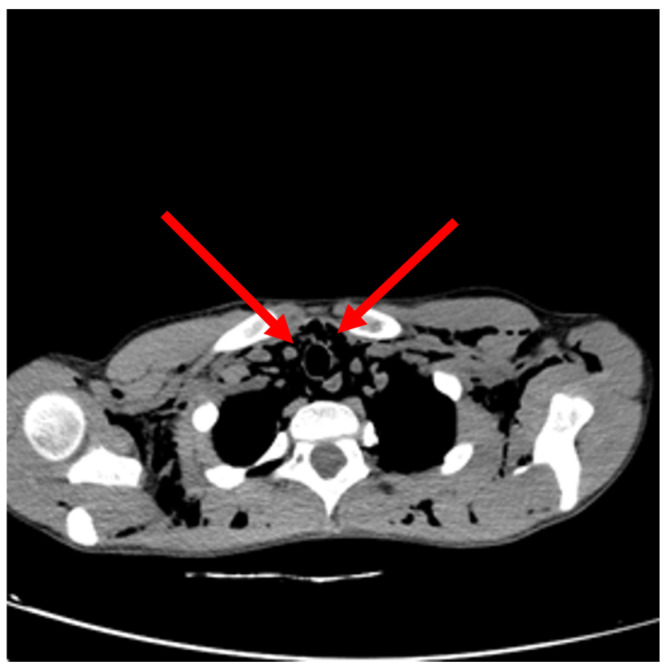
Chest CT (transverse section) showing pneumomediastinum (red arrows) with emphysema of the soft tissues of the neck and proximal thoracic wall and back.

**Figure 4:**
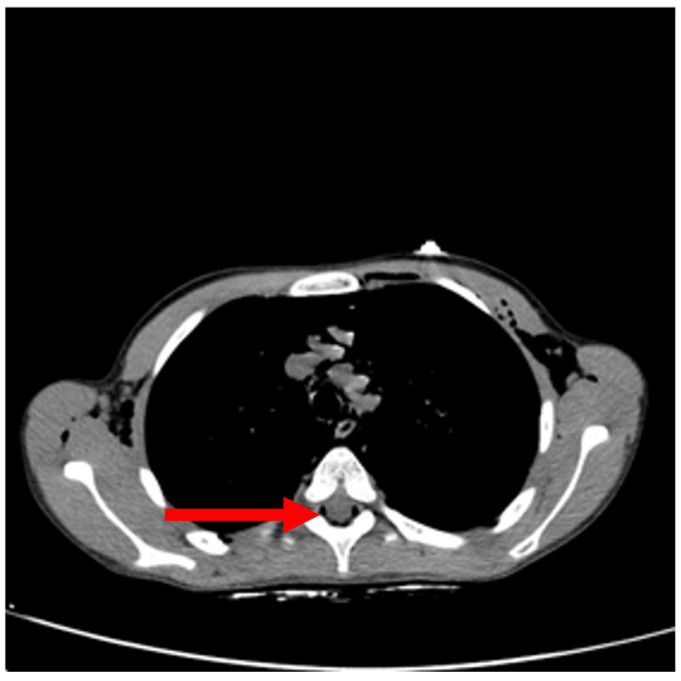
Chest CT (transverse section) showing small gas collections (red arrow) within the spinal canal.

## Data Availability

Data are unavailable due to patients’ privacy and ethical restrictions.

## Abbreviations

The following abbreviations are used in this manuscript:

A1AT	Alpha-1 antitrypsin
CT	Computed tomography
